# Functional and morphological evolution of remnant pancreas after resection for pancreatic adenocarcinoma

**DOI:** 10.1097/MD.0000000000007495

**Published:** 2017-07-14

**Authors:** Shin-Young Park, Keun-Myoung Park, Woo Young Shin, Yun-Mee Choe, Yoon-Seok Hur, Keon-Young Lee, Seung-Ik Ahn

**Affiliations:** Department of Surgery, Inha University School of Medicine, Incheon, Republic of Korea.

**Keywords:** adenocarcinoma, atrophy, glucose, pancreatectomy, residual volume

## Abstract

Functional and morphological evolution of remnant pancreas after resection for pancreatic adenocarcinoma is investigated.

The medical records of 45 patients who had undergone radical resection for pancreatic adenocarcinoma from March 2010 to September 2013 were reviewed retrospectively. There were 34 patients in the pancreaticoduodenectomy (PD) group and 10 patients in the distal pancreatectomy (DP) group. One patient received total pancreatectomy. The endocrine function was measured using the glucose tolerance index (GTI), which was derived by dividing daily maximum serum glucose fluctuation by daily minimum glucose. Remnant pancreas volume (RPV) was estimated by considering pancreas body and tail as a column, and head as an ellipsoid, respectively. The pancreatic atrophic index (PAI) was defined as the ratio of pancreatic duct width to total pancreas width. Representative indices of each patient were compared before and after resection up to 2 years postoperatively.

The area under receiver operating characteristic curve of GTI for diagnosing DM was 0.823 (95% confidence interval, 0.699–0.948, *P* < .001). Overall, GTI increased on postoperative day 1 (POD#1, mean ± standard deviation, 1.79 ± 1.40 vs preoperative, 1.02 ± 1.41; *P* = .001), and then decreased by day 7 (0.89 ± 1.16 vs POD#1, *P* < .001). In the PD group, the GTI on POD#14 became lower than preoperative (0.51 ± 0.38 vs 0.96 ± 1.37; *P* = .03). PAI in the PD group was significantly lower at 1 month postoperatively (0.22 ± 0.12 vs preoperative, 0.38 ± 0.18; *P* < .001). In the PD group, RPV was significantly lower at 1 month postoperatively (25.3 ± 18.3 cm^3^ vs preoperative, 32.4 ± 20.1 cm^3^; *P* = .02), due to the resolution of pancreatic duct dilatation. RPV of the DP group showed no significant change. GTI was negatively related to RPV preoperatively (*r* = –0.317, *P* = .04), but this correlation disappeared postoperatively (*r* = –0.044, *P* = .62).

Pancreatic endocrine functional deterioration in pancreatic adenocarcinoma patients may in part be due to pancreatic duct obstruction and dilatation caused by the tumor. After resection, this proportion of endocrine insufficiency is corrected.

## 1 Introduction

Surgical removal provides the best survival benefit for patients with pancreatic cancer.^[1–3]^ However, pancreatectomy is not without risks, which include pancreatic functional insufficiencies.^[4]^ Although ideally as much pancreatic parenchyma should be preserved as possible to maintain good endocrine and exocrine pancreatic functions, sometimes only a small volume of pancreas is left after radical resection, and concerns regarding pancreatic insufficiency develop especially when a tumor is accompanied by parenchymal atrophy.^[4–6]^ Most studies published so far on the functional and morphological evolution of remnant pancreas after resection had been conducted on patients with diverse disease entities that differ in the degrees of pancreatic duct dilatation,^[5,7–11]^ which can adversely affect pancreas function.^[12]^ Furthermore, the method usually used to evaluate pancreas endocrine function was developed for diabetes mellitus (DM),^[10]^ without consideration of the lower levels of blood glucose governed by glucagon, an important counter-regulatory hormone in glucose metabolism.^[13]^ The aim of this study was to examine the functional and morphological evolution of remnant pancreas after resection for pancreatic adenocarcinoma by developing a model based on readily available clinical data. We tried to devise a new index for pancreatic endocrine functional assessment and evaluated morphological changes of remnant pancreas by approximating it to simple geometric solid figures in accord with appearances on abdominal computed tomography (CT) images.^[14]^ The correlations between these parameters and their evolutions after surgical resection were analyzed and the implications were sought.

## 2 Patients and methods

### 2.1 Patients

From March 2010 to September 2013, a total of 46 consecutive patients with pancreatic adenocarcinoma underwent pancreatic resection with radical intent at our institute, and of these, 45 were included in this study—the excluded patient developed pancreatic cancer from an intraductal papillary mucinous neoplasm. In addition, 1 patient that had undergone total pancreatectomy was excluded from the evolutional analysis. Medical records and CT images of the enrolled patients were obtained and reviewed retrospectively. Data were collected preoperatively and until the first detection of recurrence, distant metastasis, death, or 2 years after the operation. The patients were divided into 2 groups, the pancreaticoduodenectomy group (the PD group; n = 34) and the distal pancreatectomy with splenectomy group (DP group; n = 10). The PD group was divided into 2 subgroups according to pancreaticoenteric anastomotic methods; the pancreaticojejunostomy subgroup (PJ subgroup; n = 29) and the pancreaticogastrostomy subgroup (the PG subgroup; n = 5), irrespective of pylorus preservation. In general, PG was performed when the pancreatic head cancer had been extended to the left of superior mesenteric vein, rendering conventional PJ technically difficult. The authors have decided that this study can be considered exempt from the institutional review board oversight, because this research collected existing data and the individual subjects cannot be identified directly.

### 2.2 Development of a new index for pancreatic endocrine function

DM was defined according to the criteria suggested by the American Diabetes Association.^[15]^ Using these criteria, patients were classified based on the preoperative presence or absence of DM. In patients with unstable serum glucose (SG) or with a preoperative history of DM, SG was checked 4 to 6 times daily, preoperatively and postoperatively until SG had stabilized after resumption of oral intake. Based on the assumption that pancreatic endocrine function is reflected by daily serum glucose fluctuation, we defined related parameters as follows:

SGmax: Daily maximum serum glucose level, mg/dL
SGmin: Daily minimum serum glucose level, mg/dL
ΔSG: Daily variation in SG, defined as SGmax – SGmin. In patients whose SG was stable enough to warrant no more than 1 sampling, ΔSG (mg/dL) was defined as the difference between sampled SG and 90 mg/dL.
Ireq: Daily requirements of exogenous insulin equivalents administered to maintain SG within normal ranges, irrespective of resulting SG, as measured in the international unit (IU).
Glucose tolerance index (GTI) = ΔSG/SGmin.

### 2.3 Functional evolution of remnant pancreases

The functional evolution of pancreatic endocrine function was estimated using GTI trends. Patient GTIs were checked preoperatively and at 1, 7, 14 days, and 1, 2, 3, 6, 12, 18, 24 months after the operation. When GTI values were not available for specific times, values the nearest to the study points were allocated.

### 2.4 Morphological evolution of remnant pancreases

**Figure 1:**
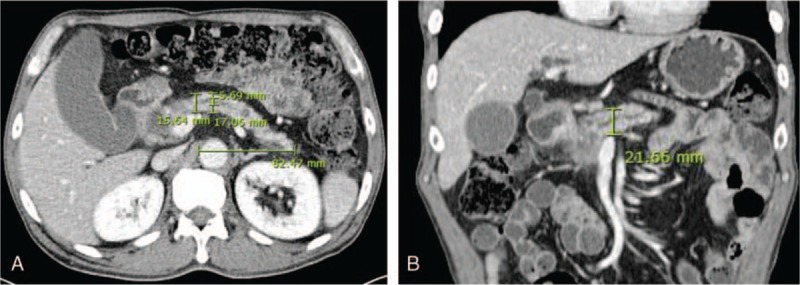
Measurements of total pancreatic width (17.06 mm), pancreatic duct width (5.69 mm), antero-posterior diameter (15.64 mm), and latero-lateral length (82.47 mm) of the pancreas body and tail in a patient with cancer of the pancreatic head (A). The cranio-caudal diameter (21.66 mm) was measured in the coronal view (B).

**Figure 2:**
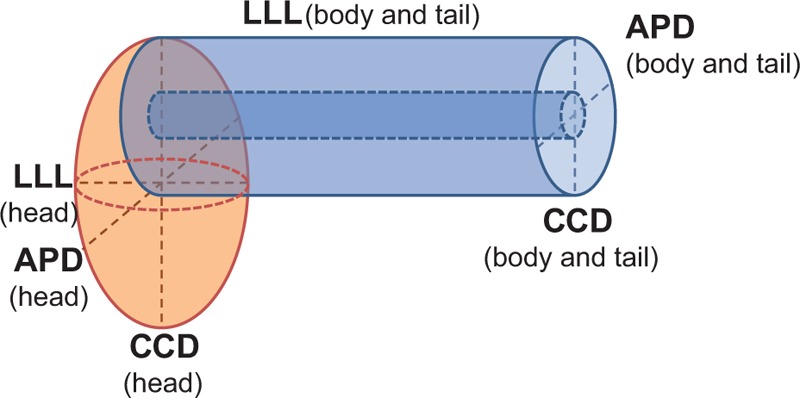
Simulation of pancreatic head, body, and tail using solid figures. APD = antero-posterior diameter, CCD = cranio-caudal diameter, LLL = latero-lateral length.

Morphological evolution was investigated using 2 indices, pancreatic atrophic index (PAI) and remnant pancreatic volume (RPV). In the PD group, PAI was defined as the pancreatic duct width (PDW) divided by total pancreatic width (TPW), as measured in the CT axial view anterior to the aorta (Fig. 1A).^[16]^ When a tumor occupied the area, PAI was measured at the nearest pancreatic parenchyma possible. In the DP group, PAI could not be measured because the main pancreatic duct within the remaining pancreas head was not dilated. Antero-posterior diameter (APD) and latero-lateral length (LLL) were measured from CT images in the axial view (Fig. 1A), and cranio-caudal diameter (CCD) was measured in the coronal view (Fig. 1B). RPV was approximated by simulating pancreas head to an ellipsoid, and body and tail to a cylinder (Fig. 2). Of note, LLL of the pancreatic body and tail was defined as the distance from the future resection plane to the distal end, and not the actual length of the remnant pancreas. The calculations were as follows.

RPV of the pancreas head = 4π/3 × APD/2 × CCD/2 × LLL/2 (cm^3^)
RPV of the pancreas body and tail = π × APD/2 × CCD/2 × LLL (cm^3^)
Corrected RPV (cRPV) = pancreas parenchymal volume = RPV – main pancreatic duct volume  = RPV × {1 – (PDW/TPW)^2^} = RPV × {1−(PAI)^2^} (cm^3^)

In the DP group, the main pancreatic duct volume of the remnant pancreas head was neglected, and cRPV was assumed to be identical to RPV. The PAI and RPV were measured preoperatively, at 1, 3, 6 months postoperatively, and 6 monthly up to 2 years. All measurements were performed using the m-view 5.4 software (Marosis Technologies Inc., Seoul, Korea).

### 2.5 Statistical analysis

Descriptive data were presented using median and range for continuous variables, and number and percent for categorical variables. The Mann–Whitney *U* test was used to compare continuous variables, and chi-square test or Fisher's exact test was used to analyze categorical variables. The relevance of GTI with respect to the presence of preoperative DM was validated by receiver operating characteristic (ROC) curve analysis. Wilcoxon's signed rank test was used for intragroup evolutional analysis. Correlation between GTI and RPV was analyzed using Pearson correlation coefficients (r). Missing data were handled by listwise deletion. The analyses were performed using IBM SPSS Statistics for Windows, Version 19.0 (IBM Corp. Armonk, NY), and a *P* value < .05 was considered statistically significant.

## 3 Results

### 3.1 Demographic data

Demographic data are presented in Table 1. Median age of all 45 patients was 64.0 years (range, 45.0–89.0 years). There were 28 men and 17 women; a male to female ratio of 1.6: 1. Median follow-up was 12.0 months (range, 1.0–57.0 months), and 24 patients (53.3%) had preoperative DM. Twenty-six patients (57.8%) received oral hypoglycemic agent (OHA) and/or insulin preparations preoperatively, whereas 27 patients (60.0%) received postoperatively. Sixteen patients (35.5%) developed grades A or B pancreatic fistula postoperatively, as defined by the International Study Group on Pancreatic Fistula (ISGPF).^[17]^ One patient (2.9%) in the PD group died of bleeding related to grade C pancreatic fistula, and overall operative mortality was 2.2%. One patient (2.2%) had T1N1 pancreatic adenocarcinoma according to the American Joint Committee on Cancer staging system,^[18]^ 11 patients (24.4%) had T3N0, and 33 patients (73.3%) had T3N1 disease. None of the demographic characteristics differed between the PD and DP groups. Table 2 shows the number of eligible patients at each study points and the reasons for nonparticipation.

**Table 1:**
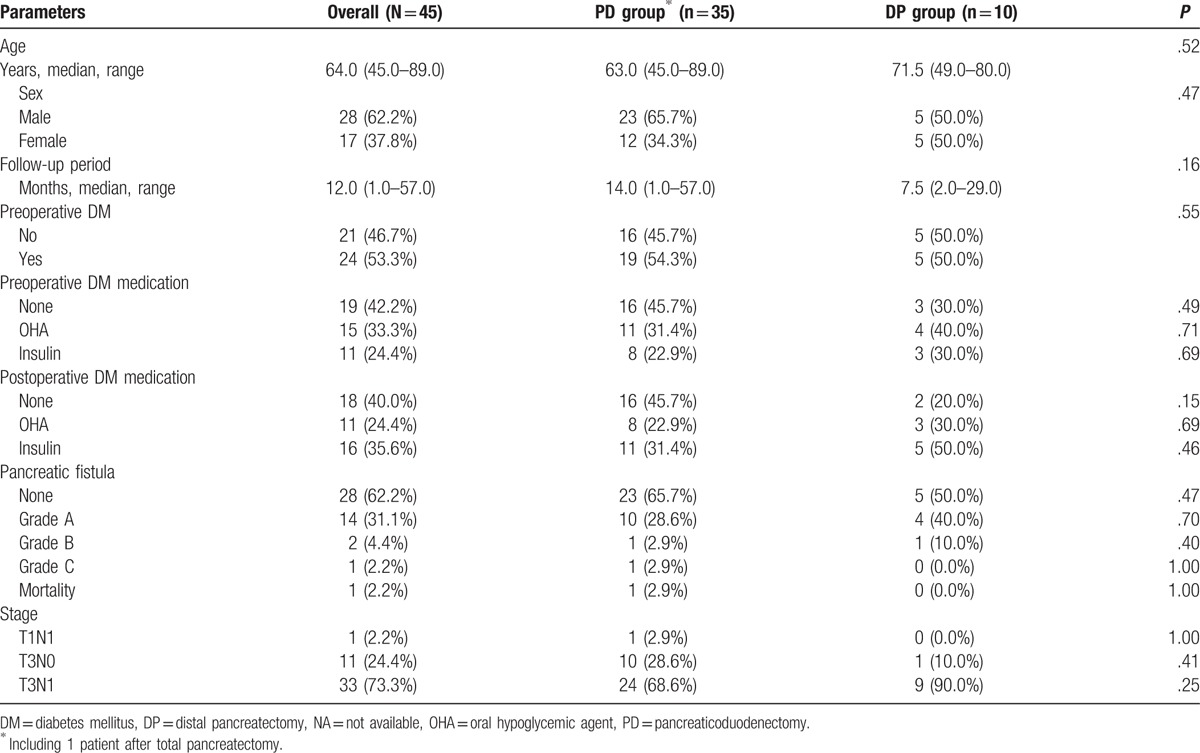
Demographic data.

**Table 2:**
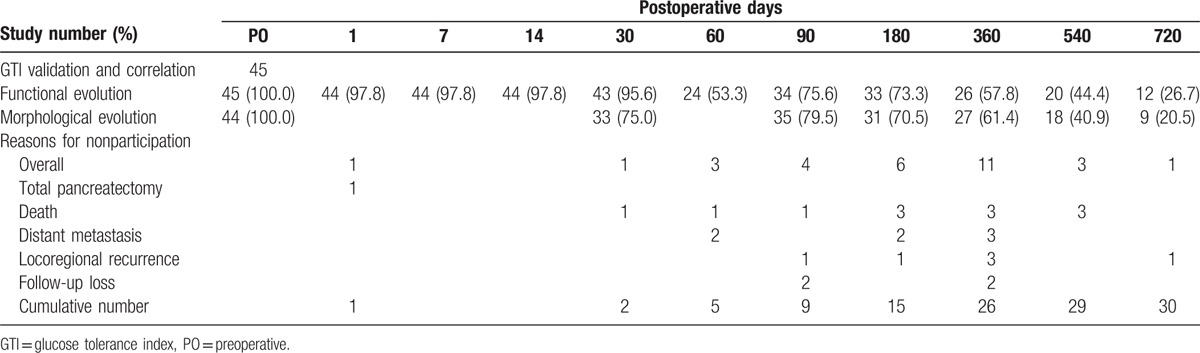
Number of eligible participants.

### 3.2 Index for pancreatic endocrine function

**Figure 3:**
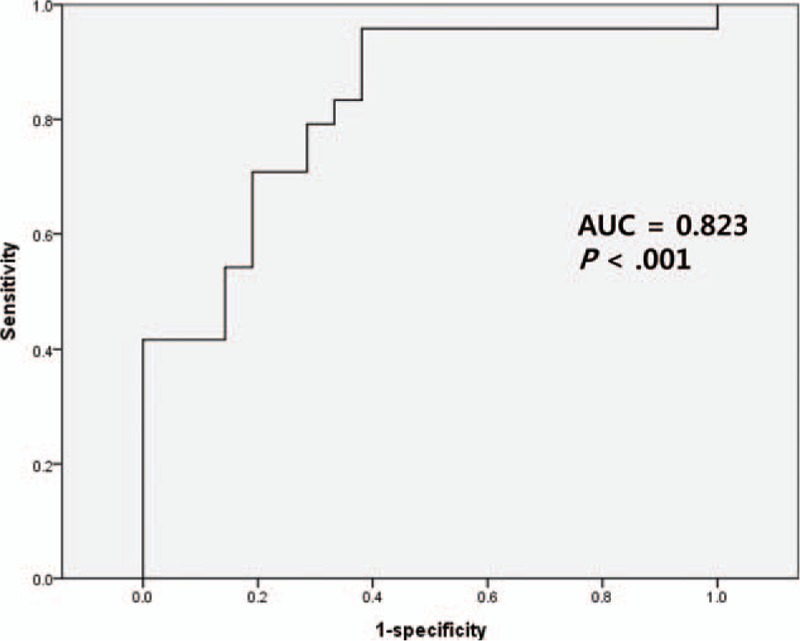
Receiver operating characteristic curve of glucose tolerance index for the diagnosis of diabetes mellitus. AUC = area under ROC curve, ROC = receiver operating characteristic.

Preoperative SGmax, SGmin, ΔSG, and Ireq were significantly different in patients with or without DM (Table 3). Of the possible combinations of these parameters, we arbitrarily defined GTI as ΔSG/SGmin. The area under the ROC curve of GTI for diagnosing DM was 0.823 (95% confidence interval, 0.699–0.948, *P* < .001; Fig. 3). When a cut-off value of 0.5 was used, the sensitivity and specificity of GTI for diagnosing DM were 79.2% and 66.7%, respectively.

**Table 3:**
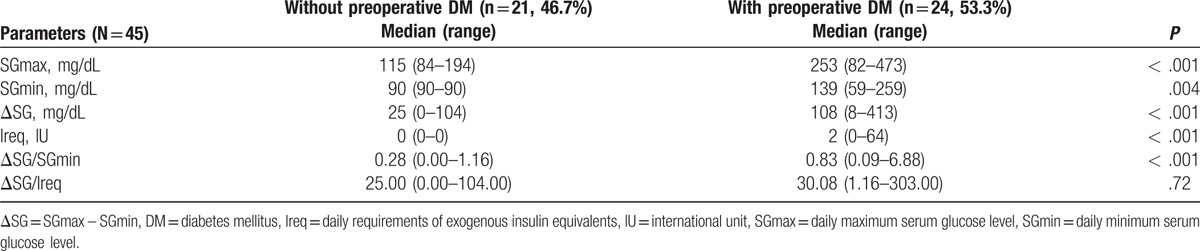
Tested preoperative parameters relevant to preoperative DM.

### 3.3 Functional evolution of remnant pancreas

One patient (2.2%) in the PD group showed overt steatorrhea during the immediate postoperative period, which improved on pancreatic enzyme supplementation. Two other patients in the PD group (5.9%) and 1 in the DP group (10%) complained of frequent greasy loose stools postoperatively. These conditions also subsided during follow-up after medication.

**Figure 4:**
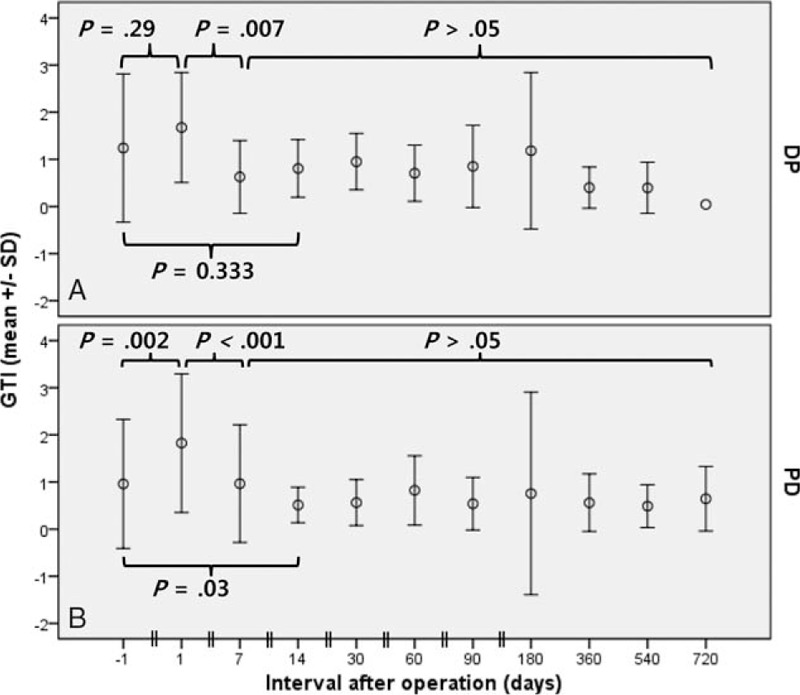
Evolution of glucose tolerance index (GTI) in diatal pancreatectomy (A) and pancreaticoduodenectomy (B) groups. Case numbers are not shown (refer to Fig. 6). DP = distal pancreatectomy, GTI = glucose tolerance index, PD = pancreaticoduodenectomy, SD = standard deviation.

Preoperative (Baseline) GTI of the 44 study subjects was 1.02 ± 1.41 (mean ± SD, standard deviation). On the first postoperative day (POD#1), GTI increased significantly (1.79 ± 1.40, *P* = .001; vs baseline), and then decreased to its preoperative value by POD#7 (0.89 ± 1.16, *P* < .001; vs POD#1). In the DP group, GTI did not change significantly thereafter (*P* > .05, Fig. 4A). However, in the PD group, the GTI on POD#14 became lower than at baseline (0.51 ± 0.38 vs 0.96 ± 1.37; *P* = .03), and remained relatively unchanged thereafter (*P* > .05; Fig. 4B). Regarding intergroup comparisons, GTI values were similar in the PD and DP groups throughout the entire study period (*P* > .05, data not shown).

### 3.4 Morphological evolution of remnant pancreas

**Figure 5:**
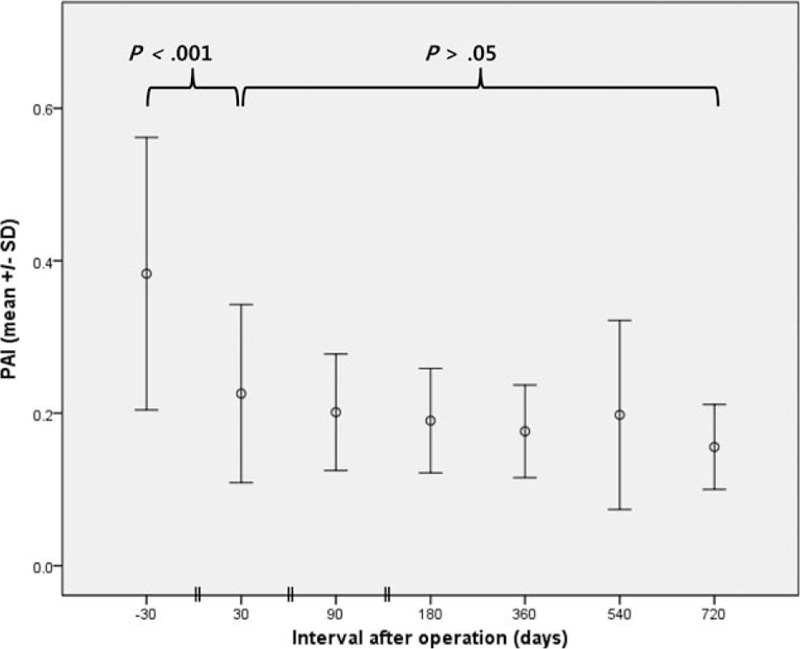
Evolution of pancreas atrophic index (PAI) in patients after pancreaticoduodenectomy. Case numbers are not shown (refer to Fig. 6). PAI =  pancreas atrophic index, SD = standard deviation.

**Figure 6:**
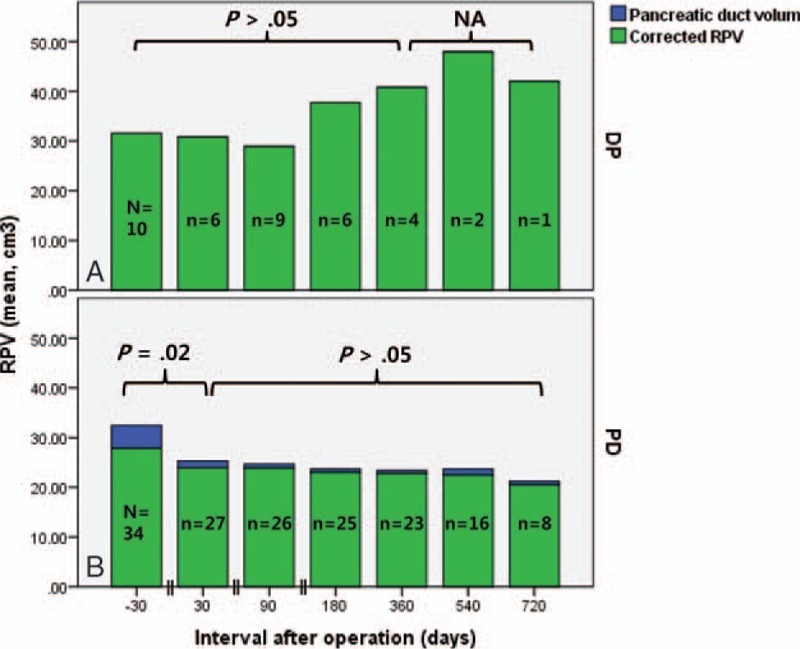
Evolution of remnant pancreatic volume (RPV) in patients after distal pancreatectomy (A) and pancreaticoduodenectomy (B). Case numbers are presented in each column. DP = distal pancreatectomy, NA = not available, PD = pancreaticoduodenectomy, RPV = remnant pancreatic volume.

Preoperative PAI in the PD group was 0.38 ± 0.18, and this decreased to 0.22 ± 0.12 (*P* < .001; vs baseline) at 1 month postoperatively, and remained stable thereafter (Fig. 5). In the DP and PD groups, preoperative RPV values were 31.6 ± 5.8 cm^3^ and 32.4 ± 20.1 cm^3^, respectively. At 1 month postoperatively, RPV in the DP group was not changed (30.8 ± 12.5 cm^3^, *P* = .92; vs baseline, Fig. 6A), whereas RPV in the PD group was significantly lower than at baseline (25.3 ± 18.3, *P* = .02, Fig. 6B). The decrease in RPV of the PD group was the result of reduced pancreatic duct volume, as evidenced by a nonsignificant change in cRPV at 1 month (27.9 ± 19.9, *P* = .33; vs baseline). In both groups, RPV remained relatively unchanged thereafter (*P* > .05).

### 3.5 Correlations between functional and morphological evolutions

**Figure 7:**
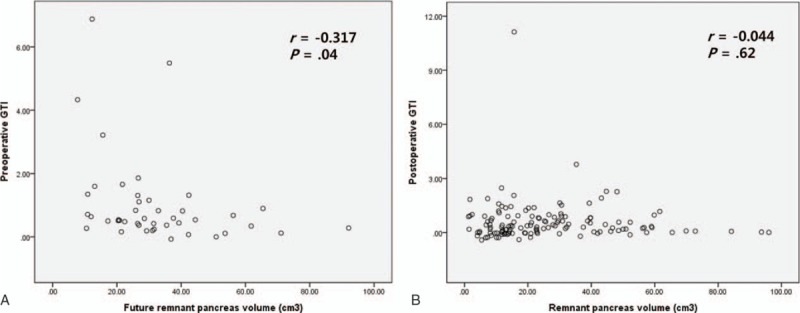
Correlations between the preoperative glucose tolerance index (GTI) and future remnant pancreats volume (A), and between postoperative GTI and remnant pancreatic volume (B). GTI = glucose tolerance index.

Preoperatively, a moderate negative linear correlation was observed between GTI and RPV (Pearson's correlation coefficient *r* = –0.317; *P* = .04) (Fig. 7A). However, this correlation disappeared postoperatively, irrespective of time after operation (*r* = –0.044, *P* = .62, Fig. 7B). Subgroup analysis showed that only the PD showed a correlation between preoperative GTI and RPV (*r* = –0.386, *P* = .02; DP group, *r* = 0.146, *P* = .69).

## 4 Discussion

This study was undertaken to examine the functional and morphological evolution of remnant pancreas after resection by developing a model based on readily available clinical data. To achieve this, we devised a new index “the glucose tolerance index” for pancreatic endocrine functional assessment. Our findings suggest pancreatic endocrine functional deterioration in pancreatic adenocarcinoma patients is in part due to tumor burden and pancreatic duct obstruction and dilatation and show that after resection, this proportion of pancreatic endocrine dysfunction can be reversed due to the correction of pancreatic duct dilatation.

The laboratory evaluations of pancreatic exocrine function involve measuring fecal fat, elastase-1, and/or chymotrypsin levels.^[7,19]^ However, because we usually do not perform fecal analysis at our institute and the retrospective nature of this study, exocrine insufficiency could not be quantitatively determined. Nevertheless, none of the enrolled patients had clinically intractable steatorrhea. Although some studies have claimed pancreas volume is correlated with pancreatic exocrine function,^[19]^ our results suggest overt pancreatic exocrine insufficiency is uncommon after standard pancreas resection for pancreatic adenocarcinoma.^[8]^

Usually pancreas endocrine insufficiency is diagnosed based on the presence of DM.^[4,20,21]^ A diagnosis of DM is by definition based on elevated serum glucose levels,^[15]^ which in turn is governed by insulin. Furthermore, the presence of DM is a nominal variable, which has limitations to analyze quantitatively. The pancreas endocrine function can also be evaluated using serum peptide C, glycosylated hemoglobin (HbA1c), or directly measured serum insulin levels.^[7,20]^ However, these parameters are not checked routinely, involve additional cost, and more importantly, focus on insulin and its activity, which represent only 1 of the 2 arms of serum glucose regulation. To assess pancreatic endocrine function properly, the effect of glucagon, a counter regulatory hormone of insulin, must also be considered.^[4,8,13]^ The action of glucagon can be considered a mirror image of that of insulin. Glucagon controls lower levels of serum glucose, as evidenced by the exacerbations of daily glucose fluctuations after total pancreatectomy (pancreatogenic DM), as compared to the diabetic patients in whom the glucagon secretion is preserved.^[4,22,23]^ We assumed that the end-result of the complex glucose control networks were daily serum glucose fluctuations, which were quantified using ΔSG in the present study. By dividing ΔSG by the daily minimum glucose level (the definition of GTI), we tried to enhance ΔSG and differentiate patients with identical ΔSG values but different glucagon activity. Another important factor of serum glucose regulation is exogenous hypoglycemic agents. However, it has been reported that exogenous insulin supplementation and oral hypoglycemic medications affect serum glucose usually a 1-sided manner and would not significantly alter ΔSG were it not for counter regulatory hormones.^[24]^ Of note, a lowering of GTI does not necessarily mean that blood glucose has decreased. Rather, it should be interpreted as an indicator of improved glucose control, and although DM may develop or worsen postoperatively, GTI reduction indicates the condition is easier to control, presumably through the preserved glucagon function.

Pancreatic volume can be measured by CT volumetry.^[20]^ However, the procedure is time-consuming and requires additional software.^[12]^ Djuric-Stefanovic et al^[14]^ proposed a simpler method for measuring the pancreatic volume by treating the pancreatic head as a column and the body and tail as a square pillar. In the present study, we likened the pancreatic head to an ellipsoid and the body and the tail to a cylinder. Our method tended to underestimate actual pancreas volumes, but calculated dimensions were comparable to previous reports,^[20,25]^ except pancreas body length, which was shorter because we measured the length perpendicular to the resection plane.^[14]^ Recently, Yoo et al^[9]^ reported the evolution of RPV during the 12 months following PD in patients with diverse periampullary tumors, and compared RPVs between patients after external and internal drainage. Although they did not mention the degree of pancreatic duct dilatation, their results showed that after PD, RPV values decreased progressively with time presumably due to pancreatic atrophy. However, it is unclear whether this decrease in RPV values was due to the resolution of pancreatic duct dilatation or the wasting of remnant parenchyma. Lemaire et al^[7]^ reported that in their series of 17 patients who had undergone PD and PG, pancreatic parenchymal thicknesses decreased and main pancreatic ducts dilated significantly after surgery. Their findings regarding main pancreatic duct evolution contradict ours possibly because of the method of pancreaticoenteric anastomosis, PG, which current study involves only 5 cases. However, they did not include pancreatic adenocarcinoma, and thus preoperative pancreatic duct dilatation was not likely.^[26]^ Also, Kim et al^[27]^ showed that pancreatic duct sizes progressively increased in the invagination group but not in the duct-to-mucosa group after PD. In view of the facts that they used a duct-to-mucosa technique in patients with duct dilated to >3 mm in diameter, and that their data included 24 (17%) patients with pancreatic carcinoma, it is probable that postoperative pancreatic duct dilatation in their series was limited to those with periampullary diseases other than cancer of the pancreatic head. The present study shows that the reduction in RPV after PD was due to a decrease in PAI, and hence, to a decrease in pancreatic duct volume, as was confirmed by the relatively unchanged corrected RPV values of patients with adenocarcinoma of the pancreatic head. As for the morphologic evolution in a DP group, Phillip et al^[25]^ reported serial increases in pancreatic head volumes in their series of 24 patients. They suggested that increases in pancreatic head volume were due to regeneration, which mandates further investigations be undertaken to differentiate hypertrophy and simple swelling. In the present study, the DP group showed the same increasing trend in pancreas head volume, but too few cases were enrolled to achieve statistical significance.

Pancreatic resection has been reported to predispose new-onset DM and worsen glucose control after partial or total pancreatectomy and that the percentage of pancreatic parenchymal loss is roughly correlated with postoperative endocrine insufficiency, as evidenced by newly developed DM and worsening of glucose intolerance,^[4,5,20]^ although there are contradictory reports.^[28]^ In general, an RPV of 20% is considered the minimum requirement to avoid clinically relevant DM, provided that pancreas parenchyma is free of diffuse pathology after PD or DP.^[12]^ Recently, Burkhart et al^[10]^ reported 48% of patients experienced aggravation of preexisting DM after PD as compared with 26% after DP, and that 18% of patients developed DM after PD as compared with 31% after DP. This seemingly disproportional aggravation of DM in patients after PD and DP could be due to the different distributions of pancreatic α- and β-cells, which secrete glucagon and insulin, respectively.^[10]^ When we studied the evolution of pancreas endocrine function using GTI instead of DM, GTI was observed to increase significantly during the immediate postoperative period, which probably reflects acute glucose tolerance deterioration due to operation, inflammation, and infection.^[29]^ At 7 days postoperatively, when most patients resumed oral intake, indulged in active exercise, and systemic inflammation was under control, GTI decreased significantly, and subsequently remained stable. The favorable GTI profile after the immediate postoperative period was observed mainly in the PD group, presumably because more pancreas parenchyma is preserved after PD^[14]^ and more α-cells remain.^[10]^ However, these explanations inadequately explain why GTI improved versus preoperative values after pancreatic resection, which inevitably involves loss of pancreatic volume. It could be that after PD, pancreatic duct obstruction caused by pancreatic cancer is relieved, and thus remaining distal pancreas is decompressed. Pancreatic adenocarcinoma is frequently accompanied by atrophy and duct dilatation distal to the tumor,^[26]^ and duct obstruction can interfere with pancreatic endocrine function and can cause DM.^[12,14]^ There are reports that preoperative DM improved after PD in some patients,^[10,12,30]^ supporting our data that the acute pancreatic duct dilatation caused by obstruction by pancreatic cancer is reversible. Another mechanical change that accompanies is the removal of tumor mass, which can secrete diabetogenic agent.^[30]^ However, we could not observe the improvement of GTI after DP, although the tumors had been removed in both PD and DP groups. The causal relationship between pancreatic cancer and DM is unclear, although some evidence indicates DM caused by pancreatic cancer may be reversible after surgical resection.^[30]^ In the present study, the prevalence of preoperative DM in all patients was 53.3%, which was more than twice as higher than values reported on mixed disease entities.^[9,10,12]^ Considering that baseline serum glucose was measured during the immediate preoperative period, it is likely that so-called acute or new-onset DM cases caused by pancreatic cancer, whether by duct obstruction or tumor-related diabetogenic material, were included in the present study.^[19,30]^ Our results show that such cases may exhibit improved pancreas endocrine function after removal of the tumor burden and amelioration of duct obstruction by surgical resection,^[8]^ as evidenced by the evolution of GTI and PAI, and the disappearance of the correlation between GTI and RPV postoperatively.^[28]^ DM is the hallmark of pancreatic endocrine insufficiency, and is a multifactorial entity that embraces volume-related factors, such as insulin and glucagon secreting abilities, and cancer-related factors, such as tumor burden and duct obstruction and dilatation. The present study shows the latter can be corrected by surgical resection, at the cost of pancreas volume. Surgery for pancreatic adenocarcinoma should be approached differently from other diseases requiring pancreas resection, in terms of remnant pancreas volume and the risk of developing postoperative endocrine insufficiency, because DM arising from pancreatic adenocarcinoma is different.

The major limitation of this study comes from the design itself, which is retrospective in nature. Clinical data were sometimes missing at certain study points, due to individual patient's follow-up schedule not being standardized as well as poor prognosis of pancreatic adenocarcinoma. A limited number of relevant cases made parametric analysis not applicable, decreasing the power of statistical analyses. Further studies with better-designed protocol and more cases are warranted. Comparison between pancreatic head cancers with distal pancreatic duct obstruction and other periampullary cancers without pancreatic duct dilatation can further clarify the results of this study. Also, GTI should be refined to evaluate pancreatic endocrine function more accurately, including other related variables such as oral hypoglycemic agents and exogenous insulin supplements.

In conclusion, the pancreatic endocrine function can be evaluated using the glucose tolerance index, which is defined as the ratio of daily serum glucose fluctuation and daily minimum glucose level, and thus, is not based wholly on the status of diabetes mellitus. In patients with pancreatic adenocarcinoma, the pancreatic endocrine function is further compromised by pancreatic duct obstruction and dilatation caused by the tumor, and this proportion of pancreatic endocrine dysfunction can be reversed by standard pancreatic resection.
